# Perspectives of health practitioners and adults who regained weight on predictors of relapse in weight loss maintenance behaviors: a concept mapping study

**DOI:** 10.1080/21642850.2021.2014332

**Published:** 2021-12-26

**Authors:** Eline M. Roordink, Ingrid H.M. Steenhuis, Willemieke Kroeze, Mai J.M. Chinapaw, Maartje M. van Stralen

**Affiliations:** a Department of Health Sciences, Faculty of Science, Vrije Universiteit Amsterdam, Amsterdam Public Health Research Institute, Amsterdam, The Netherlands; b Department Care for Nutrition and Health, School of Nursing, Christian University of Applied Sciences, Ede, The Netherlands; c Department of Public and Occupational Health, Amsterdam Public Health Research Institute, Amsterdam UMC, Vrije Universiteit Amsterdam, Amsterdam, The Netherlands

**Keywords:** Relapse, weight loss maintenance, dietary behavior, physical activity, concept mapping

## Abstract

**Background:**

Preventing people from relapsing into unhealthy habits requires insight into predictors of relapse in weight loss maintenance behaviors. We aimed to explore predictors of relapse in physical activity and dietary behavior from the perspectives of health practitioners and persons who regained weight, and identify new predictors of relapse beyond existing knowledge.

**Methods:**

We used concept mapping to collect data, by organizing eight concept mapping sessions among health practitioners (N=39, five groups) and persons who regained weight (N=21, three groups). At the start of each session, we collected participants’ ideas on potential predictors. Subsequently, participants individually sorted these ideas by relatedness and rated them on importance. We created concept maps using principal component analysis and cluster analysis.

**Results:**

43 predictors were identified, of which the majority belonged to the individual domain rather than the environmental domain. Although the majority of predictors were mentioned by both stakeholder groups, both groups had different opinions regarding their importance. Also, some predictors were mentioned by only one of the two stakeholder groups. Practitioners indicated change in daily structure, stress, maladaptive coping skills, habitual behavior, and lack of self-efficacy regarding weight loss maintenance as most important recurrent (mentioned in all groups) predictors. Persons who regained weight indicated lifestyle imbalance or experiencing a life event, lack of perseverance, negative emotional state, abstinence violation effect, decrease in motivation and indulgence as most important recurrent predictors.

**Conclusions:**

For several predictors associations with relapse were shown in prior research; additionally, some new predictors were identified that have not been directly associated with relapse in weight loss maintenance behaviors. Our finding that both groups differed in opinion regarding the importance of predictors or identified different predictors, may provide an opportunity to enhance lifestyle coaching by creating more awareness of these possible discrepancies and including both points of view during coaching.

## Introduction

For people with obesity, losing five percent of their body weight can already have major health benefits, such as improved body composition and metabolic function (Magkos et al., 2016). However, maintaining weight loss by making sustainable changes in physical activity and dietary behavior has proven to be challenging. On average, 30-35% of the lost weight is regained in the first year after weight loss, and after this year weight gain generally continues (Turk et al., 2009). To maintain weight loss, relapse, i.e. a breakdown or failure in a person's attempt to change their lifestyle and maintain their target behavior, has to be prevented (Marlatt & George, 1984). To effectively prevent people from relapsing, insight is required into the predictors of relapse in weight loss maintenance behaviors. The current study therefore aims to explore predictors of relapse in weight loss maintenance behaviors from the perspectives of two stakeholder groups: health practitioners who supervise and coach adults in their weight loss process and adults who recently lost weight and experienced one or more relapses.

Insight into predictors of relapse is provided by theoretical models such as Marlatt’s Relapse Prevention Model (RPM) (Marlatt & Gordon, 1985) and the self-regulation theory (Baumeister, Heatherton, & Tice, 1994). Although the RPM was originally developed for relapse prevention in drug abstinence, it has been successfully applied to a wide range of health behaviors, including obesity (Dombrowski et al., 2012; Marlatt & Donovan, 2005). According to the RPM, risk of relapse occurs when an individual enters a so-called high-risk situation, i.e. any situation that poses a threat to the individual's sense of control. When someone lacks effective coping responses while entering the high-risk situation, a decrease in self-efficacy will occur and the probability of lapse, i.e. a slip or mistake, will arise. If this person also has positive outcome expectancies towards the old habitual behavior, the chance of lapsing will increase even more (Marlatt & George, 1984). Whether this first lapse is followed by a complete relapse depends on the cause to which the individual attributes the lapse. A negative attribution of the cause is called the abstinence violation effect: if the individual attributes the lapse to their own personal failure (e.g. guilt, shame) and to stable, internal factors beyond their control (e.g. no willpower), the risk of relapse increases (Larimer & Marlatt, 2004; Marlatt & George, 1984).

According to the self-regulation theory, the abstinence violation effect is one of the possible lapse-activated responses that contributes to self-regulation failure (Baumeister & Heatherton, 1996). Within this theory, lapse-activated responses are described as a class of behaviors that emerge after an initial failure of self-regulation, a lapse. These responses cause a minor breakdown in self-control, often activating factors that prevent the reassertion of self-control, resulting in an acceleration of the breakdown. Often, it is not the lapse itself, but the subsequent breakdown in self-control that has the most severe effects on behavioral maintenance (Baumeister & Heatherton, 1996; Baumeister, Schmeichel, & Vohs, 2007).

Apart from theories, insight into predictors of relapse can be obtained from previous studies; such as the recent literature review by Roordink and colleagues (Roordink et al., 2021) on the predictors of lapse and relapse in physical activity and dietary behavior, based on 37 prospective studies. Regarding physical activity, this study found a higher risk of relapse for people with a lower self-efficacy, fewer behavioral processes of change (i.e. covert and overt activities to modify behavior), and less self-regulation. For dietary behavior, it found that people with lower self-efficacy had a higher risk of relapsing (Roordink et al., 2021). However, the review also showed that there is still insufficient evidence for most predictors of relapse. As of yet, current literature still lacks an in-depth understanding of key stakeholders’ personal perspectives on relapse after weight loss. These key stakeholders include adults attempting weight loss and health practitioners.

Perspectives from these key stakeholders could provide new and important insights from daily practice on predictors of relapse in weight loss maintenance behaviors, which can inform future relapse prevention interventions. We therefore aimed to identify predictors of relapse in physical activity and dietary behavior, from the perspective of health practitioners who coach individuals during their weight loss process and the perspective of individuals who have experienced relapses themselves. In addition, we aimed to identify possible new predictors of relapse in physical activity and dietary behavior beyond existing knowledge, using concept mapping. Concept mapping is a structured methodology combining qualitative and quantitative methods to integrate group thought and perspectives about a particular topic, in order to produce a conceptual framework (Burke et al., 2005). Concept mapping has been applied successfully to address complex issues in health care (W. Trochim & Kane, 2005).

## Methods

### Design

**Figure 1.**
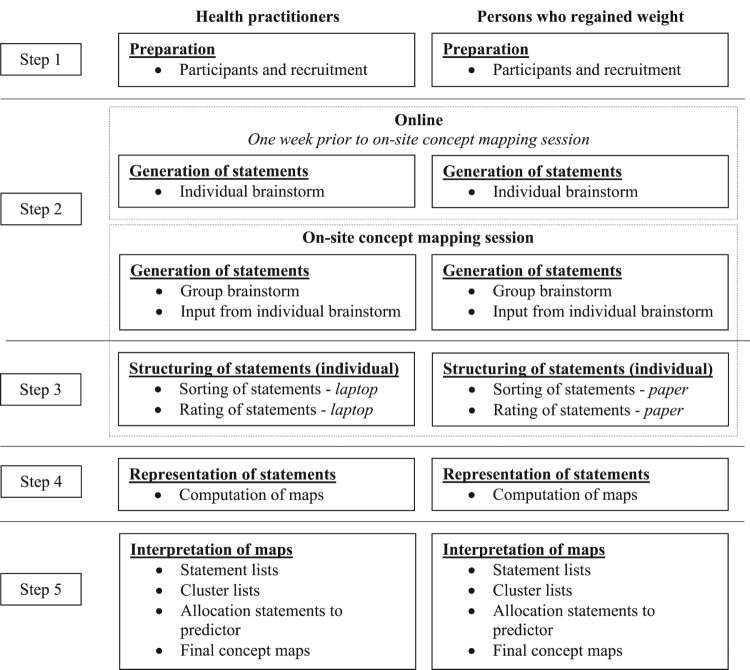
The concept mapping process. Adapted from ‘An introduction to concept mapping for program planning and evaluation’ by W. Trochim, 1989, Evaluation and Program Planning, 12, 1-16.

A concept mapping study was conducted with data collection between November 2017 and July 2018. The following steps from the process of concept mapping (Burke et al., 2005; W. M. Trochim, 1989) were applied: 1. preparation, 2. generation of statements, 3. structuring of statements, 4. representation of statements, and 5. interpretation of maps, see Figure 1.

### Step 1. Preparation

Conceptualization is at its best when it includes a diversity of relevant people, to ensure that a variety of viewpoints is considered (W. M. Trochim, 1989). Therefore, our study included two stakeholder groups: 1. health practitioners who supervise and coach adults in losing weight, and 2. persons who regained weight: adults who recently lost weight and experienced at least one relapse (determined by the persons who regained weight themselves). Within these stakeholder groups, we aimed for a diverse sample, e.g. multiple professions and demographically heterogeneous persons who regained weight. The health practitioners included dietitians, weight loss consultants and lifestyle coaches. These are the three types of professionals that people in the Netherlands most commonly turn to for weight loss counseling and support. Inclusion criterion for all was the ability to speak Dutch. For the persons who regained weight, it was having recently (< one year) lost weight through behavioral change instead of a self-reported crash diet (i.e. consuming minimal levels of food to lose a lot of weight in a short period of time) or bariatric surgery.

Per stakeholder group, we aimed for four subgroups across the Netherlands, including 7–10 participants per subgroup or until saturation was reached, which is in accordance with the concept mapping literature (Kane & Trochim, 2007). Health practitioners were recruited by a call for participation on LinkedIn, an online network for professionals. Health practitioners were able to sign up individually or with a group of colleagues. The persons who regained weight were recruited via multiple strategies: through health practitioners participating in our study, a call for participation on social media (Facebook, LinkedIn), direct contact with local walking groups, and flyers at GP practices, health centers and the Vrije Universiteit Amsterdam. Additionally, we made use of our personal network and snowball sampling. Interested participants could contact the researchers by email, after which concept mapping groups were composed. When participants registered individually, groups were composed based on proximity of residence. Participants received a certificate of participation (health practitioners only), a factsheet with the study findings, and a small financial compensation. After agreeing to participate, online informed consent was obtained. The study was conducted according to the ethical standards declared in the Declaration of Helsinki. Following the criteria of the Dutch Medical Research Involving Human Subjects Act, our study complies with the Code of Ethics of the Faculty of Science of the Vrije Universiteit Amsterdam; therefore, our study did not require further evaluation by the Research Ethics Review Committee

### Step 2. Generation of statements

One week before the on-site concept mapping session, in which a subgroup got together, each participant received a link to an online questionnaire per email. The questionnaire was developed for this study and contained questions on age, sex, educational level, and profession (health practitioners only), see Supplementary file 1. Based on the standard classification of the Central Bureau of Statistics Netherlands (CBS), educational level was categorized into three levels: low level of education (primary education and lower general secondary education), middle level of education (higher general secondary education, pre-university education, and secondary vocational education), and high level of education (bachelor’s degree, master’s degree, and doctoral degree; (Statistics Netherlands, n.d.)). Furthermore, participants were encouraged to share all their ideas on perceived predictors of relapse in weight loss maintenance behaviors, by responding to the following focus statement: ‘*A relapse in physical activity and/or dietary behavior is caused by..*.’. The purpose of a focus statement is to give the brainstorm an area of focus, in order to address the primary research question (Burke et al., 2005). When losing weight, behavioral changes in physical activity and dietary behavior are often combined, resulting in their determinants often being intertwined (e.g. general motivation, self-efficacy) (Dombrowski, Knittle, Avenell, Araújo-Soares, & Sniehotta, 2014; Kwasnicka, Dombrowski, White, & Sniehotta, 2017, p. 2019). Therefore, we decided to combine both weight loss maintenance behaviors in one focus statement. The focus statement was pre-tested and evaluated during the concept mapping sessions, which confirmed its feasibility. Health practitioners were asked to address the focus statement on population level, i.e. based on their professional experience, whereas the persons who regained weight did this based on personal experience. All collected ideas, hereafter referred to as statements, were used as input for a group brainstorm during the on-site concept mapping session, which was facilitated by two researchers (ER with MVS or WK). During the brainstorm, participants were encouraged to come up with more statements and clarification was asked for statements that were unclear, resulting in an extensive list of unique statements per subgroup.

### Step 3. Structuring of statements

Following the brainstorm during the on-site concept mapping session, participants structured the statements by individually sorting them into piles of related statements, i.e. categories. To complete this task, health practitioners used the online software program Ariadne (Ariadne, n.d.), whereas the persons who regained weight received the statements on printed cards. Due to the settings of the software program, a minimum of three and a maximum of ten categories were required, with at least two statements per category. A miscellaneous category was prohibited. Participants were instructed to individually place each statement in a category and name the categories. Subsequently, each participant individually rated the importance of each statement on a 5-point Likert scale, ranging from very unimportant (1) to very important (5), by answering the following question: ‘*How important is each specific statement in predicting a relapse in physical activity and/or dietary behavior?’*.

The on-site concept mapping session for the health practitioners lasted 1.5 h and the session for the persons who regained weight lasted two hours. The difference in sorting and rating methods between stakeholder groups (online vs print) was based on recommendations given by the health practitioners, who believed sorting statements on paper would be easier than doing it online. As participants came up with new statements during the brainstorm session, a portable label printer was used to print new cards for the persons who regained weight. For the health practitioners, all new statements were directly entered into the online software. After the sessions with the persons who regained weight, one of the researchers (ER) entered the data in Ariadne. Once entered in Ariadne, data could no longer be traced back to individuals.

### Step 4. Representation of statements (statistical analysis)

Using the Ariadne-software, the structured statements were transformed, per subgroup, into a matrix representing the similarity between statements for each participant. A higher number signifies a higher conceptual similarity between ideas. All the individual matrices were then transformed into one matrix representing all the individuals in that subgroup. This matrix was used as input for the Principal Component Analysis (PCA), which translated the distance (i.e. correlations) between statements into coordinates in a multi-dimensional space (Sleddens et al., 2015). The statements were further classified by completing a cluster analysis with the coordinates of the statements. These clusters were graphically presented in a PCA plot. Statements that were closer to each other in the plot were sorted together more often (and vice versa). Also, the mean importance for each statement and cluster was calculated. Additionally, IBM SPSS Statistics 26 was used to perform descriptive analysis on participants demographics.

### Step 5. Interpretation of the maps

By default, Ariadne computed eight clusters. The research team discussed whether fewer or more clusters would represent participants’ statements better, by evaluating the coherence between statements in each cluster. After defining the final number of clusters, each statement within a cluster was evaluated and allocated to a perceived predictor (e.g. the statement ‘lack of motivation’ was allocated to the perceived predictor ‘motivation’). Subsequently, the research team named all clusters, thereby keeping the names given by the participants in consideration. Within the groups, each cluster represented multiple perceived predictors; this made it impossible to do a group comparison on cluster level. We therefore analyzed our results on predictor level instead of cluster level, which is in accordance with former concept mapping literature (Hidding, Chinapaw, & Altenburg, 2018). To do so, the mean importance of each perceived predictor was calculated based on the overall mean importance ratings of the underlying statements.

## Results

### Participants

A total of 39 health practitioners participated in this study (39 women, Mage = 48.2 years, SDage = 9.9), distributed over five groups, see Table 1. For the groups consisting of persons who regained weight, 21 participants participated in this study (18 women, Mage = 53.5 years, SDage = 8.9), distributed over three groups, see Table 1. Saturation was reached for both stakeholder groups, meaning no new statements came up during the final concept mapping sessions.

**Table 1.** Demographics and characteristics of health practitioners and persons who regained weight.

	**Health practitioners** **(N = 39)**	**Persons who regained weight** **(N = 21)**
**Gender (N)**		
Male	0	3
Female	39	18
**Age (years)**		
Mean (SD)	48.2 (9.9)	53.5 (8.9)
Educational level (N)*		
Low	3	3
Middle	12	13
High	24	5
**Profession (N)**		
Dietitian	14	-
Weight loss consultant	19	-
Lifestyle coach	5	-
Other	1	-

*Low level of education: primary education and lower general secondary education; Middle level of education: higher general secondary education, pre-university education, and secondary vocational education; High level of education: bachelor’s degree, master’s degree, and doctoral degree.

### Health practitioners

The subgroups of health practitioners produced between 66 and 79 unique statements, sorted into 38 different predictors, including 32 individual predictors and six environmental predictors, see Table 2. Some of the perceived predictors were behavior specific (e.g. weather barriers), which is indicated in Table 2 with superscripts. There were 17 recurrent (mentioned in all health practitioner groups) perceived predictors of relapse in weight loss maintenance behaviors: change in daily structure; stress; maladaptive coping skills; habitual behavior; lack of self-efficacy regarding weight loss maintenance; decrease in motivation; lifestyle imbalance or experiencing a life event; lack of perseverance; negative emotional state; non-supportive physical environment; goal disengagement; lack of social support; non-effective weight loss strategy; obstructing beliefs about lifestyle change; abstinence violation effect; perceived financial barriers; and perceived weather barriers.

**Table 2.** Perceived predictors of relapse and importance rating as indicated by health practitioners and persons who regained weight.

		**Health practitioners (N = 39)**					**Persons who regained weight (N = 21)**				
	Example of statement within perceived predictor	Mean importance rating per group*					Mean importance rating of all groups	Mean importance rating per group			Mean importance rating of all groups
**Perceived predictors**		**P1**	**P2**	**P3**	**P4**	**P5**		**W1**	**W2**	**W3**	
**Individual characteristics**											
Change in daily structure	*‘changes in living situation, e.g. vacation, changes daily structure’*	3.5	4.4	4.4	3.8	4.6	4.1	-	-	-	-
Stress	*‘experiencing stress’*	4.0	4.1	4.1	3.6	4.6	4.1	4.0	3.8	-	3.9
Maladaptive coping skills^a^	*‘finding comfort in food’*	3.9	3.9	4.4	4.5	3.7	4.1	4.0	4.1	-	4.1
Habitual behavior	*‘strong former unhealthy habits’*	3.5	4.1	4.3	4.0	4.4	4.1	-	-	-	-
Lack of self-efficacy regarding weight loss maintenance	*‘lack of faith in yourself’*	3.5	4.1	4.3	4.3	4.2	4.1	3.7	-	-	3.7
Decrease in motivation	*‘not feeling like dieting or exercising anymore’*	3.6	4.3	3.7	4.4	3.9	4.0	3.9	3.7	3.6	3.7
Lifestyle imbalance or experiencing a life event	*‘setbacks in life’*	3.1	3.9	4.4	4.3	4.2	4.0	3.5	4.2	3.9	3.9
Lack of perseverance	*‘lack of discipline’*	4.2	3.6	3.2	4.3	4.3	3.9	3.9	3.8	3.6	3.8
Negative emotional state	*‘not being at ease with yourself’*	3.0	4.1	3.0	3.9	4.6	3.7	3.9	3.8	3.5	3.7
Goal disengagement	*‘it takes too long to see results’*	3.7	3.6	2.8	4.3	4.0	3.7	4.2	3.4	-	3.8
Maladaptive weight loss strategy	*‘starting a diet that is not realistic to maintain’*	3.5	4.0	3.6	3.8	3.4	3.7	-	3.0	3.5	3.3
Obstructing beliefs about lifestyle change	*‘seeing losing weight as an ongoing battle’*	3.4	4.0	3.3	3.6	3.6	3.6	3.4	3.6	3.5	3.5
Abstinence violation effect	*‘today I failed, so I give up’*	3.3	3.7	3.1	3.7	3.7	3.5	3.5	3.8	3.9	3.7
Lack of self-knowledge regarding needs and difficulties	*‘not knowing their own pitfalls’*	4.6	4.6	-	4.6	3.8	4.4	-	-	-	-
Unrealistic initial goal setting	*‘setting the bar too high’*	3.8	4.1	3.8	4.2	-	4.0	4.0	-	-	4.0
Limited cognitive capacity	*‘lack of ability to be flexible, e.g. to vary in diet’*	3.6	3.5	4.3	-	3.4	3.7	3.1	-	3.5	3.3
Physiological state^a^	*‘being hungry’*	3.4	3.6	-	3.3	3.5	3.5	-	3.4	-	3.4
Physiological barriers^b^	*‘having an injury’*	2.7	3.3	-	3.1	3.1	3.1	3.7	3.2	2.7	3.2
Perceived time barriers	*‘having too little time to exercise’*	2.9	3.0	2.5	-	3.4	3.0	3.2	2.8	2.6	2.9
Low self-value	*‘lack of self-esteem’*	-	4.4	4.4	-	4.0	4.3	-	-	-	-
Lack of resilience	*‘lack of resilience’*	4.0	-	3.9	4.5	-	4.1	-	-	-	-
No sense of urgency to lose weight	*‘not seeing the urgent need of losing weight’*	3.7	4.5	-	4.0	-	4.1	-	-	3.6	3.6
Lack of maintenance goal setting	*‘after the weight loss goal is reached, the attention drops’*	3.4	-	4.1	3.5	-	3.7	-	4.4	4.1	4.3
Lack of self-monitoring	*‘not being aware of a relapse’*	3.6	-	3.8	3.5	-	3.6	-	-	-	-
Perceived general barriers	*‘unable to cook dinner’*	2.7	3.6	-	3.4	-	3.2	-	-	-	-
Unrealistic outcome expectancy	*‘too high expectations’*	4.0	-	-	-	3.9	4.0	-	-	2.8	2.8
Excuses	*‘seeking excuses’*	-	-	2.4	4.0	-	3.2	-	4.3	-	4.3
Lack of self-control	*‘lack of self-control’*	-	-	-	4.5	-	4.5	-	-	-	-
General emotional state	*‘falling back into old patterns due to emotional events’*	-	-	4.4	-	-	4.4	-	-	4.6	4.6
Locus of control	*‘feelings of having no influence’*	3.7	-	-	-	-	3.7	-	-	-	-
Impulsivity	*‘character: difficult to control yourself’*	-	3.4	-	-	-	3.4	-	-	-	-
Positive emotional state	*‘celebrating with dinner’*	-	3.1	-	-	-	3.1	-	-	3.4	3.4
Craving^a^	*‘feeling that you must snack in the evening’*	-	-	-	-	-	-	-	4.2	3.8	4.0
Indulgence^a^	*‘food as a reward’*	-	-	-	-	-	-	3.6	3.7	3.8	3.7
Lack of knowledge^a^	*‘too much and contradictory information about nutrition in the media’*	-	-	-	-	-	-	3.6	2.7	3.4	3.2
Rationalization	*‘already having a hard enough time’*	-	-	-	-	-	-	-	2.8	-	2.8
**Environmental characteristics**											
Non-supportive physical environment	*‘tempting environment at work and at home’*	3.6	3.5	3.7	3.4	4.2	3.7	3.9	3.4	3.4	3.6
Lack of social support	*‘ceasing of a sport buddy’*	3.2	3.5	3.5	4.3	3.9	3.7	3.4	3.1	3.0	3.2
Perceived financial barriers	*‘not having enough financial resources’*	2.9	3.1	2.5	2.5	2.9	2.8	-	-	-	-
Perceived weather barriers^b^	*‘the weather is bad’*	2.3	2.4	2.3	2.8	2.7	2.5	3.3	3.9	2.0	3.1
Social pressure^a^	*‘group pressure, e.g. at a party’*	2.9	3.2	3.6	-	3.9	3.4	-	-	-	-
Social norm	*‘don’t want to say no when someone made an effort’*	2.7	-	3.5	3.5	3.7	3.4	-	3.3	3.8	3.6
Tempting social environment^a^	*‘seeing other people eat’*	-	-	-	-	-	-	3.7	3.2	3.6	3.5

Not applicable (-), indicates a perceived predictor was not mentioned during the concept mapping session within this group.

*Mean importance rating per group: P indicates ‘practitioners’; W indicates ‘persons who regained weight’. Numbers indicate the different groups (e.g. W1 = weight regain group 1).

^a^ Perceived predictor is specifically aimed at dietary behavior.

^b^ Perceived predictor is specifically aimed at physical activity.

Of the recurrent perceived predictors, the following five predictors received the highest ratings (mean group ratings all above 4.0 on a scale from 1 (very unimportant) to 5 (very important)): change in daily structure, stress, maladaptive coping skills, habitual behavior, and lack of self-efficacy regarding weight loss maintenance. Although not mentioned by every health practitioner group, lack of self-knowledge regarding needs and difficulties, low self-value, lack of resilience, no sense of urgency to lose weight, lack of self-control, and general emotional state also received high ratings (mean group ratings all above 4.0).

### Persons who regained weight

The subgroups of persons who regained weight produced between 35 and 81 statements, sorted into 31 different predictors, including 26 personal and five environmental predictors, see Table 2. There were 13 recurrent (mentioned in all weight regain groups) perceived predictors of relapse in weight loss maintenance behaviors: obstructing beliefs about lifestyle change; negative emotional state; indulgence; lack of knowledge; lifestyle imbalance or experiencing a life event; decrease in motivation; lack of perseverance; physiological barriers; perceived time barriers; perceived weather barriers; tempting social environment; lack of social support; and abstinence violation effect.

Of the recurrent perceived predictors, the following six predictors received the highest ratings (mean group ratings all above 4.0): lifestyle imbalance or experiencing a life event, lack of perseverance, negative emotional state, abstinence violation effect, decrease in motivation, and indulgence. Although not mentioned by every weight regain group, maladaptive coping skills, lack of maintenance goal setting, excuses, general emotional state, and craving also received high ratings (mean group ratings all above 4.0).

Although the majority of perceived predictors were mentioned by both stakeholder groups, opinions differed slightly between the two. Compared to the persons who regained weight, the health practitioners rated different perceived predictors were rated as most important. In addition, a few perceived predictors were mentioned by all practitioner groups, but not by the weight regain groups: change in daily structure, perceived financial barriers, and habitual behavior. Conversely, perceived predictors that were mentioned by all weight regain groups, but not by the health practitioners were: tempting social environment, indulgence, and lack of knowledge.

## Discussion

This concept mapping study aimed to identify predictors of relapse in weight loss maintenance behaviors from the perspectives of health practitioners and persons who regained weight. Additionally, we aimed to identify possible new predictors of weight loss maintenance behaviors beyond existing knowledge. According to the health practitioners, the most important recurrent predictors (mean group ratings above 4.0) were: change in daily structure, stress, maladaptive coping skills, habitual behavior, and lack of self-efficacy regarding weight loss maintenance. According to the persons who regained weight, the most important recurrent predictors (mean group ratings between 3.7 and 3.9) were: lifestyle imbalance or experiencing a life event, lack of perseverance, negative emotional state, abstinence violation effect, decrease in motivation and indulgence.

Looking at these most important recurrent predictors, our results are partly coherent with findings from quantitative relapse studies. A recent systematic review of prospective studies on predictors of relapse in dietary behavior and physical activity confirmed self-efficacy and coping skills as predictors (Roordink et al., 2021). The abstinence violation effect has also been confirmed as predictor in the past (Carels et al., 2001; Herman & Mack, 1975; Kendzierski & Sheffield, 2000; Polivy, 1976; Polivy, Herman, Younger, & Erskine, 1979; Stetson et al., 2005). In addition, self-efficacy, coping and the abstinence violation effect are also covered in the Relapse Prevention Model (RPM) (Marlatt & Gordon, 1985). Although the RPM also covers stress, negative emotional state, indulgence, and lifestyle imbalance or experiencing a life event, further scientific evidence on their relation with relapse in physical activity and dietary behavior is lacking (Roordink et al., 2021). Previous research on factors influencing (maintenance of) physical activity and dietary behavior may provide some first indications that these factors are indeed relevant predictors of relapse in weight loss maintenance behaviors. For example, recent reviews showed that stress impairs efforts to be physically active, and stress and negative emotions unfavorably influence dietary behavior (Araiza & Lobel, 2018; Devonport, Nicholls, & Fullerton, 2019; Frayn & Knäuper, 2018; Stults-Kolehmainen & Sinha, 2014); however, evidence for an association between negative emotions and physical activity remains inconclusive (Kruk et al., 2019; Liao, Shonkoff, & Dunton, 2015). Furthermore, indulgence has been associated with unhealthy eating behavior by multiple studies, especially when the indulgence is considered justified (‘I worked so hard today, I deserve it’) (Effron, Monin, & Miller, 2013; Prinsen, Evers, & de Ridder, 2016). Despite the indicated association between lifestyle imbalance and relapse according to the RPM, studies assessing this potential association are, to our knowledge, currently lacking.

Our stakeholders also indicated decrease in motivation, habitual behavior, change in daily structure and lack of perseverance as most important perceived predictors. As scientific evidence on their association with relapse in weight loss maintenance behaviors is once again lacking (Roordink et al., 2021), general theories on motivation and behavior change and research on factors influencing (maintenance of) physical activity and dietary behavior may provide more insight. First, the self-determination theory indicates intrinsic motivation as an important factor for behavior change maintenance: behavior change is more likely to be maintained if the new behavior is perceived as personally relevant and reflects an individual's values (Kwasnicka, Dombrowski, White, & Sniehotta, 2016). As most individuals start behavior change attempts when their motivation is high, a decrease in motivation over time could lead to a relapse into previous behavior (Kwasnicka et al., 2016). The association between intrinsic motivation and health behavior change has been shown present in the area of physical activity and dietary behavior (Amireault, Godin, & Vézina-Im, 2013; Ng et al., 2012; Teixeira, Carraça, Markland, Silva, & Ryan, 2012). Second, habits, defined as a form of automaticity triggered by situational cues and enacted with little conscious awareness, play an important role in people’s failure to adopt and maintain healthy behavior (Orbell & Verplanken, 2010; Wood & Neal, 2016). Therefore, both breaking and creating habits are central to behavior change (Wood & Neal, 2016). Research shows eating habits can be directly activated by environmental cues, without the activation of preferences and goals (Neal, Wood, Wu, & Kurlander, 2011; Rothman, Sheeran, & Wood, 2009). The formation of new habits is triggered by stable features in the environment (Wood, Quinn, & Kashy, 2002); this may explain why a change in an individual’s daily rhythm**,** often related to a change of environment (e.g. going on holiday), can lead to a relapse into previous behavior. Last, for perseverance, described as the continuation of a goal-directed action in spite of obstacles (Peterson & Seligman, 2004), knowledge about its potential association with behavior change is currently lacking. More research is recommended to confirm the relation between these perceived predictors and relapse in weight loss maintenance behaviors.

With regard to our second aim, based on the perspectives from health practitioners and persons who regained weight there are a number of predictors that are newly identified in this study. These perceived predictors all received high scores (all mean group ratings above 4.0 according to at least one of the two stakeholder groups), but, to the best of our knowledge, have not yet been directly associated with relapse in weight loss maintenance behaviors in prior research (Roordink et al., 2021). Perceived predictors that we believe have not been previously researched in relation to relapse in weight loss maintenance behaviors are change in daily structure, lifestyle imbalance or experiencing a life event, lack of self-knowledge regarding needs and difficulties, unrealistic initial goal setting, lack of maintenance goal setting, low self-value, lack of resilience, excuses, and no sense of urgency to lose weight. For some perceived predictors its relationship with relapse in weight loss maintenance behaviors has been researched before, including stress, habitual behavior, decrease in motivation, abstinence violation effect, lack of self-control, general emotional state, and craving; however, evidence to support a relationship is lacking as these predictors were only studied once or evidence remains inconclusive (Carels, Cacciapaglia, Rydin, Douglass, & Harper, 2006; Carels et al., 2001; Forman et al., 2017; Manasse et al., 2018a, 2018b; McKee, Ntoumanis, & Taylor, 2014; Meule, Richard, & Platte, 2017; Mooney, Burling, Hartman, & Brenner-Liss, 1992; Schlundt, Virts, Sbrocco, Pope-Cordle, & Hill, 1993; Simkin & Gross, 1994; Stiggelbout, Hopman-Rock, Crone, Lechner, & Van Mechelen, 2006). Future research should further investigate the predictive value of these perceived predictors in relation to relapse in weight loss maintenance behaviors.

### Differences between stakeholder groups

Although the majority of perceived predictors were mentioned by both stakeholder groups, they had different opinions regarding their importance. In addition, the two stakeholder groups also differed regarding how often certain perceived predictors were mentioned; a few predictors were mentioned by all practitioner groups, but not by the persons who regained weight, and vice versa. A possible explanation for these differences is that health practitioners base their knowledge on their experience with many clients, and therefore generate and rate statements based on the average person (seeing ‘the bigger picture’). The persons who regained weight may have generated and rated statements based on their own experiences, leaving more room for diversity. This emphasizes the importance of including multiple stakeholders to gather diverse views and form a more complete picture. Furthermore, results show that both stakeholder groups predominantly rate individual factors as most important perceived predictors of relapse. However, previous research indicates that environmental factors, such as a tempting environment, also influence relapse (Roordink et al., 2021). It is possible that individuals do not know or like to admit they are being influenced by their social or physical environment. In addition, the influence of the social or physical environment is often felt in combination with individual factors (e.g. not being able to cope with the social pressure at a party), which might make environmental factors more distal and therefore harder to recall. This remoteness of environmental factors is also reflected in the so-called fundamental attribution error, which is defined as ‘the tendency for attributors to underestimate the impact of situational factors and to overestimate the role of dispositional factors in controlling behavior’ (Ross, 1977). Participants’ greater focus on individual factors could furthermore be stimulated by the current stigma surrounding overweight and obese individuals and the notion that they are to blame for their weight (Puhl & Heuer, 2010).

### Strengths and limitations

Our study provides new insights into the predictors of relapse in weight loss maintenance behaviors from the perspective of key stakeholders, contributing to a more in-depth understanding of relapse with the help of personal perspectives and experiences from daily practice. The focus on both health practitioners and persons who regained weight perspectives is an important strength of this study, providing insight from multiple points of view. Furthermore, the concept mapping method allows multiple points of view in each group of stakeholders to be integrated whilst taking the relative importance of each statement into account, using valid statistical methods (Rosas & Kane, 2012). However, there are also some limitations worth mentioning. During the statement sorting process, we noticed that both stakeholder groups found it challenging to start sorting the statements into piles. This was mainly because they were not allowed to create more than 10 piles, due to the settings of the software program. As the stakeholders identified a wide range of predictors, it might have been easier to place the statements into better fitting categories if they were allowed to create more piles. Furthermore, as each cluster represented multiple perceived predictors, which appeared during the interpretation of the maps, it seems that the data may have been too complex to base the results entirely on a mathematical model. Therefore, expert opinions and existing theoretical categories were needed to be able to analyze the results on a predictor level instead of cluster level. Also, although we had our reasons to combine physical activity and dietary behavior, there is a possibility that some (behavior specific) predictors are missed due to the combining of behaviors. Last, a more diverse sample would have been preferred: males were underrepresented, and the majority of the persons who regained weight had a middle educational level.

### Future research

Several recommendations for future research can be made. First, as we wanted to keep the generation of statements feasible and non-confusing for the participants, we formulated one focus statement in which the predictors of physical activity and dietary behavior were combined and no distinction between lapse and relapse was made. Although, based on the underlying statements, the majority of the indicated perceived predictors apply to both physical activity and dietary behavior, some of the perceived predictors were behavior specific. For example, ‘maladaptive coping skills’ was specifically aimed at dietary behavior, whereas ‘perceived weather barriers’ was specifically aimed at physical activity. Future research could further investigate potential differences between the predictors of relapse in physical activity and dietary behavior, and between lapse and relapse.

Second, for several predictors scientific evidence for a direct association with relapse in weight loss maintenance behaviors is lacking in prior research. These include for example lack of perseverance and lack of resilience. Therefore, to examine whether the identified perceived predictors in this study indeed predict relapse in weight loss maintenance behaviors, a larger prospective study is recommended. We suggest an ecological momentary assessment (EMA) study to track experiences over time and get insight into the process of behavior change, among which lapsing and relapsing (Shiffman, Stone, & Hufford, 2008). EMA has been proven useful in measuring lapses and relapses in previous studies (Carels et al., 2006; Carels, Douglass, Cacciapaglia, & O'Brien, 2004; Carels et al., 2001; Forman et al., 2017; Latner, McLeod, O’Brien, & Johnston, 2013; McKee et al., 2014), and therefore provides an opportunity to confirm the perceived predictors identified in this study. As EMA minimizes recall bias and maximizes ecological validity, it could also be a useful design to assess whether individual predictors of relapse are indeed of higher importance than environmental predictors, or if for example the fundamental attribution error plays a part in this finding (Shiffman et al., 2008).

Last, it would be of interest to develop a theoretical framework, consisting of various predictors of relapse in weight loss maintenance behaviors and its dynamic interactions. Such a framework should not only include predictors that are known from prior models, such as Marlatt’s Relapse Prevention Model, but also predictors that have been newly identified in this study and other recent studies (Kwasnicka, Dombrowski, White, & Sniehotta, 2019; Roordink et al., 2021). For example, in this study self-value and resilience received high importance ratings, but these are not reflected in current models. We believe a theoretical framework based on the latest insights would be of added value to the field of relapse prevention and can inform future weight loss maintenance interventions.

### Practical implications

Although more research on the predictors of relapse in weight loss maintenance behaviors is recommended, careful implications for practice can be made. The differences between the predictors perceived by health practitioners and those perceived by persons who regained weight may provide an opportunity to enhance lifestyle coaching, by creating more awareness of these possible discrepancies and ensuring that both points of view are included during coaching. Clients are more likely to be satisfied and follow advice on health behavior change when they feel they have been heard and understood, and are given information they recognize as relevant to them (Gable, 2007). The identified predictors could be relevant for future weight loss interventions that prevent relapse in weight loss maintenance behaviors by combining evidence-based techniques for altering relevant changeable predictors (e.g. effective coping skills) and coping with relevant non-changeable predictors (e.g. experiencing a life event). Planning coping responses to anticipated, personal, high-risk situations helps an individual to cope with difficult situations, such as negative emotions or being tempted by their social or physical environment (Sniehotta, Schwarzer, Scholz, & Schüz, 2005). Coping planning has been shown to be an efficacious technique to promote health behavior change, especially when individuals receive support when forming coping plans (Kwasnicka, Presseau, White, & Sniehotta, 2013). Therefore, we advise health practitioners to support their clients by helping them to identify personal risk situations and formulating corresponding coping plans.

## Conclusion

To conclude, our study provides new insights into the predictors of relapse in weight loss maintenance behaviors identified as relevant by health practitioners (i.e. change in daily structure, stress, maladaptive coping skills, habitual behavior, and lack of self-efficacy) as well as persons who regained weight (i.e. lifestyle imbalance, experiencing a life event, lack of perseverance, negative emotional state, abstinence violation effect, decrease in motivation and indulgence). For several identified predictors an association with relapse in weight loss maintenance behaviors was shown in prior research and/or reflected in relapse theories. Additionally, some new predictors were identified that have not yet been directly associated with relapse in weight loss maintenance behaviors in prior research; for example, self-value and resilience. Furthermore, both stakeholder groups predominantly rated individual factors as the most important perceived predictors of relapse. Our finding that these groups sometimes identified different predictors or differed in opinion regarding the importance of the perceived predictors may provide an opportunity to enhance lifestyle coaching, by creating more awareness of these possible discrepancies and ensuring that both points of view are included during coaching. Future research could invest in developing a theoretical framework, consisting of various predictors of relapse in weight loss maintenance behaviors and its dynamic interactions, and including newly identified predictors.

## Supplementary Material

Supplemental Material

## Data Availability

The datasets used and analyzed during the current study are available from the corresponding author on reasonable request.

## References

[CIT0001] Amireault, S., Godin, G., & Vézina-Im, L.-A. (2013). Determinants of physical activity maintenance: A systematic review and meta-analyses. *Health Psychology Review*, *7*(1), 55–91.

[CIT0002] Araiza, A. M., & Lobel, M. (2018). Stress and eating: Definitions, findings, explanations, and implications. *Social and Personality Psychology Compass*, *12*(4), 1–13.

[CIT0003] Ariadne. (n.d.). Ariadne for concept mapping online. Retrieved from http://www.minds21.org/

[CIT0004] Baumeister, R. F., & Heatherton, T. F. (1996). Self-regulation failure: An overview. *Psychological Inquiry*, *7*(1), 1–15.

[CIT0005] Baumeister, R. F., Heatherton, T. F., & Tice, D. M. (1994). *Losing control: How and why people fail at self-regulation*. In: San Diego, CA: Academic Press.

[CIT0006] Baumeister, R. F., Schmeichel, B. J., & Vohs, K. D. (2007). Self-regulation and the executive function: The self as controlling agent. *Social Psychology: Handbook of Basic Principles*, *2*, 516–539.

[CIT0007] Burke, J. G., O’Campo, P., Peak, G. L., Gielen, A. C., McDonnell, K. A., & Trochim, W. M. (2005). An introduction to concept mapping as a participatory public health research method. *Qualitative Health Research*, *15*(10), 1392–1410. 16263919 10.1177/1049732305278876

[CIT0008] Carels, R. A., Cacciapaglia, H. M., Rydin, S., Douglass, O. M., & Harper, J. (2006). Can social desirability interfere with success in a behavioral weight loss program? *Psychology and Health*, *21*(1), 65–78.

[CIT0009] Carels, R. A., Douglass, O. M., Cacciapaglia, H. M., & O'Brien, W. H. (2004). An ecological momentary assessment of relapse crises in dieting. *Journal of Consulting and Clinical Psychology*, *72*(2), 341–348. 15065966 10.1037/0022-006X.72.2.341

[CIT0010] Carels, R. A., Hoffman, J., Collins, A., Raber, A. C., Cacciapaglia, H., & O'Brien, W. H. (2001). Ecological momentary assessment of temptation and lapse in dieting. *Eating Behaviors*, *2*(4), 307–321. 15001025 10.1016/s1471-0153(01)00037-x

[CIT0011] Devonport, T. J., Nicholls, W., & Fullerton, C. (2019). A systematic review of the association between emotions and eating behaviour in normal and overweight adult populations. *Journal of Health Psychology*, *24*(1), 3–24. 28810437 10.1177/1359105317697813

[CIT0012] Dombrowski, S. U., Knittle, K., Avenell, A., Araújo-Soares, V., & Sniehotta, F. F. (2014). Long term maintenance of weight loss with non-surgical interventions in obese adults: Systematic review and meta-analyses of randomised controlled trials. *BMJ*, *348*, 1–12. 10.1136/bmj.g2646 PMC4020585 25134100

[CIT0013] Dombrowski, S. U., Sniehotta, F. F., Avenell, A., Johnston, M., MacLennan, G., & Araújo-Soares, V. (2012). Identifying active ingredients in complex behavioural interventions for obese adults with obesity-related co-morbidities or additional risk factors for co-morbidities: A systematic review. *Health Psychology Review*, *6*(1), 7–32.

[CIT0014] Effron, D. A., Monin, B., & Miller, D. T. (2013). The unhealthy road not taken: Licensing indulgence by exaggerating counterfactual sins. *Journal of Experimental Social Psychology*, *49*(3), 573–578.

[CIT0015] Forman, E. M., Schumacher, L. M., Crosby, R., Manasse, S. M., Goldstein, S. P., Butryn, M. L., … Graham Thomas, J. (2017). Ecological momentary assessment of dietary lapses across behavioral weight loss treatment: Characteristics, predictors, and relationships with weight change. *Annals of Behavioral Medicine*, *51*(5), 741–753. 28281136 10.1007/s12160-017-9897-x PMC5591758

[CIT0016] Frayn, M., & Knäuper, B. (2018). Emotional eating and weight in adults: A review. *Current Psychology*, *37*(4), 924–933.

[CIT0017] Gable, J. (2007). *Counselling skills for dietitians*. Chichester: John Wiley & Sons.

[CIT0018] Herman, C. P., & Mack, D. (1975). Restrained and unrestrained eating 1. *Journal of Personality*, *43*(4), 647–660. 1206453 10.1111/j.1467-6494.1975.tb00727.x

[CIT0019] Hidding, L., Chinapaw, M., & Altenburg, T. (2018). An activity-friendly environment from the adolescent perspective: A concept mapping study. *International Journal of Behavioral Nutrition and Physical Activity*, *15*(1), 1–8. 10.1186/s12966-018-0733-x PMC6192111 30326895

[CIT0020] Kane, M., & Trochim, W. M. (2007). *Concept mapping for planning and evaluation* (Vol. 50). Thousand Oaks: Sage Publications.

[CIT0021] Kendzierski, D., & Sheffield, A. (2000). Self-schema and attributions for an exercise lapse. *Basic and Applied Social Psychology*, *22*(1), 1–8.

[CIT0022] Kruk, M., Zarychta, K., Horodyska, K., Boberska, M., Scholz, U., Radtke, T., & Luszczynska, A. (2019). What comes first, negative emotions, positive emotions, or moderate-to-vigorous physical activity? *Mental Health and Physical Activity*, *16*, 38–42.

[CIT0023] Kwasnicka, D., Dombrowski, S. U., White, M., & Sniehotta, F. (2016). Theoretical explanations for maintenance of behaviour change: A systematic review of behaviour theories. *Health Psychology Review*, *10*(3), 277–296. 26854092 10.1080/17437199.2016.1151372 PMC4975085

[CIT0024] Kwasnicka, D., Dombrowski, S. U., White, M., & Sniehotta, F. F. (2017). N-of-1 study of weight loss maintenance assessing predictors of physical activity, adherence to weight loss plan and weight change. *Psychology & Health*, *32*(6), 686–708. 28323457 10.1080/08870446.2017.1293057

[CIT0025] Kwasnicka, D., Dombrowski, S. U., White, M., & Sniehotta, F. F. (2019). ‘It’s not a diet, it’sa lifestyle’: A longitudinal, data-prompted interview study of weight loss maintenance. *Psychology & Health*, *34*(8), 963–982. 30905184 10.1080/08870446.2019.1579913

[CIT0026] Kwasnicka, D., Presseau, J., White, M., & Sniehotta, F. F. (2013). Does planning how to cope with anticipated barriers facilitate health-related behaviour change? A systematic review. *Health Psychology Review*, *7*(2), 129–145.

[CIT0027] Larimer, M. E., & Marlatt, G. A. (2004). Relapse prevention: An overview of Marlatt’s cognitive-behavioral model. In Elinore McCance-Katz H. Westley Clark (Ed.), *Psychosocial treatments* (pp. 11–28). New York: Routledge.

[CIT0028] Latner, J. D., McLeod, G., O’Brien, K. S., & Johnston, L. (2013). The role of self-efficacy, coping, and lapses in weight maintenance. *Eating and Weight Disorders-Studies on Anorexia, Bulimia and Obesity*, *18*(4), 359–366. 10.1007/s40519-013-0068-1 24078407

[CIT0029] Liao, Y., Shonkoff, E. T., & Dunton, G. F. (2015). The acute relationships between affect, physical feeling states, and physical activity in daily life: A review of current evidence. *Frontiers in Psychology*, *6*, 1–7. 26779049 10.3389/fpsyg.2015.01975 PMC4688389

[CIT0030] Magkos, F., Fraterrigo, G., Yoshino, J., Luecking, C., Kirbach, K., Kelly, S. C., … Patterson, B. W. (2016). Effects of moderate and subsequent progressive weight loss on metabolic function and adipose tissue biology in humans with obesity. *Cell Metabolism*, *23*(4), 591–601. 26916363 10.1016/j.cmet.2016.02.005 PMC4833627

[CIT0031] Manasse, S. M., Crochiere, R. J., Dallal, D. H., Lieber, E. W., Schumacher, L. M., Crosby, R. D., … Forman, E. M. (2018a). A multimodal investigation of impulsivity as a moderator of the relation between momentary elevations in negative internal states and subsequent dietary lapses. *Appetite*, *127*, 52–58. 29715502 10.1016/j.appet.2018.04.025 PMC10148240

[CIT0032] Manasse, S. M., Schumacher, L. M., Goldstein, S. P., Martin, G. J., Crosby, R. D., Juarascio, A. S., … Forman, E. M. (2018b). Are individuals with loss-of-control eating more prone to dietary lapse in behavioural weight loss treatment? An ecological momentary assessment study. *European Eating Disorders Review*, *26*(3), 259–264. 29484774 10.1002/erv.2583 PMC5916047

[CIT0033] Marlatt, G., & Gordon, J. (1985). *Relapse prevention: Maintenance strategies in the treatment of addictive behaviors*. New York: Guilford.

[CIT0034] Marlatt, G. A., & Donovan, D. M. (2005). *Relapse prevention: Maintenance strategies in the treatment of addictive behaviors*. New York: Guilford press.

[CIT0035] Marlatt, G. A., & George, W. H. (1984). Relapse prevention: Introduction and overview of the model. *British Journal of Addiction*, *79*(4), 261–273. 6595020 10.1111/j.1360-0443.1984.tb00274.x

[CIT0036] McKee, H. C., Ntoumanis, N., & Taylor, I. M. (2014). An ecological momentary assessment of lapse occurrences in dieters. *Annals of Behavioral Medicine*, *48*(3), 300–310. doi:10.1007/s12160-014-9594-y 24562984

[CIT0037] Meule, A., Richard, A., & Platte, P. (2017). Food cravings prospectively predict decreases in perceived self-regulatory success in dieting. *Eating Behaviors*, *24*, 34–38. 27987433 10.1016/j.eatbeh.2016.11.007

[CIT0038] Mooney, J. P., Burling, T. A., Hartman, W. M., & Brenner-Liss, D. (1992). The abstinence violation effect and very low calorie diet success. *Addictive Behaviors*, *17*(4), 319–324. 1502966 10.1016/0306-4603(92)90038-w

[CIT0039] Neal, D. T., Wood, W., Wu, M., & Kurlander, D. (2011). The pull of the past: When do habits persist despite conflict with motives? *Personality and Social Psychology Bulletin*, *37*(11), 1428–1437. 21859902 10.1177/0146167211419863

[CIT0040] Ng, J. Y., Ntoumanis, N., Thøgersen-Ntoumani, C., Deci, E. L., Ryan, R. M., Duda, J. L., & Williams, G. C. (2012). Self-determination theory applied to health contexts: A meta-analysis. *Perspectives on Psychological Science*, *7*(4), 325–340. 26168470 10.1177/1745691612447309

[CIT0041] Orbell, S., & Verplanken, B. (2010). The automatic component of habit in health behavior: Habit as cue-contingent automaticity. *Health Psychology*, *29*(4), 374–383. 20658824 10.1037/a0019596

[CIT0042] Peterson, C., & Seligman, M. E. (2004). *Character strengths and virtues: A handbook and classification* (Vol. 1). Oxford: Oxford University Press.

[CIT0043] Polivy, J. (1976). Perception of calories and regulation of intake in restrained and unrestrained subjects. *Addictive Behaviors*, *1*(3), 237–243.

[CIT0044] Polivy, J., Herman, C. P., Younger, J. C., & Erskine, B. (1979). Effects of a model on eating behavior: The induction of a restrained eating style 1. *Journal of Personality*, *47*(1), 100–117. 430328 10.1111/j.1467-6494.1979.tb00617.x

[CIT0045] Prinsen, S., Evers, C., & de Ridder, D. (2016). Oops I did it again: Examining self-licensing effects in a subsequent self-regulation dilemma. *Applied Psychology: Health and Well-Being*, *8*(1), 104–126. 26970112 10.1111/aphw.12064

[CIT0046] Puhl, R. M., & Heuer, C. A. (2010). Obesity stigma: Important considerations for public health. *American Journal of Public Health*, *100*(6), 1019–1028. 20075322 10.2105/AJPH.2009.159491 PMC2866597

[CIT0047] Roordink, E. M., Steenhuis, I. H. M., Kroeze, W., Schoonmade, L. J., Sniehotta, F. F., & van Stralen, M. M. (2021). Predictors of lapse and relapse in physical activity and dietary behaviour: A systematic search and review on prospective studies. Psychology & Health, doi:10.1080/08870446.2021.1981900. 34851220

[CIT0048] Rosas, S. R., & Kane, M. (2012). Quality and rigor of the concept mapping methodology: A pooled study analysis. *Evaluation and Program Planning*, *35*(2), 236–245. 22221889 10.1016/j.evalprogplan.2011.10.003

[CIT0049] Ross, L. (1977). The intuitive psychologist and his shortcomings: Distortions in the attribution process. In Leonard Berkowitz (Ed.), *Advances in experimental social psychology* (Vol. 10, pp. 173–220). New York: Academic Press.

[CIT0050] Rothman, A. J., Sheeran, P., & Wood, W. (2009). Reflective and automatic processes in the initiation and maintenance of dietary change. *Annals of Behavioral Medicine*, *38*(suppl_1), s4–s17. 19787308 10.1007/s12160-009-9118-3

[CIT0051] Schlundt, D. G., Virts, K. L., Sbrocco, T., Pope-Cordle, J., & Hill, J. O. (1993). A sequential behavioral analysis of cravings sweets in obese women. *Addictive Behaviors*, *18*(1), 67–80. 8465679 10.1016/0306-4603(93)90010-7

[CIT0052] Shiffman, S., Stone, A. A., & Hufford, M. R. (2008). Ecological momentary assessment. *Annu. Review of Clinical Psychology*, *4*, 1–32. 10.1146/annurev.clinpsy.3.022806.091415 18509902

[CIT0053] Simkin, L. R., & Gross, A. M. (1994). Assessment of coping with high-risk situations for exercise relapse among healthy women. *Health Psychology*, *13*(3), 274–277. 8055862 10.1037//0278-6133.13.3.274

[CIT0054] Sleddens, E. F., Kroeze, W., Kohl, L. F., Bolten, L. M., Velema, E., Kaspers, P., … Brug, J. (2015). Correlates of dietary behavior in adults: An umbrella review. *Nutrition Reviews*, *73*(8), 477–499. 26106126 10.1093/nutrit/nuv007 PMC4502713

[CIT0055] Sniehotta, F. F., Schwarzer, R., Scholz, U., & Schüz, B. (2005). Action planning and coping planning for long-term lifestyle change: Theory and assessment. *European Journal of Social Psychology*, *35*(4), 565–576.

[CIT0056] Statistics Netherlands. (n.d.). Classificaties. Retrieved from https://www.cbs.nl/nl-nl/onze-diensten/methoden/classificaties

[CIT0057] Stetson, B. A., Beacham, A. O., Frommelt, S. J., Boutelle, K. N., Cole, J. D., Ziegler, C. H., & Looney, S. W. (2005). Exercise slips in high-risk situations and activity patterns in long-term exercisers: An application of the relapse prevention model. *Annals of Behavioral Medicine*, *30*(1), 25–35. 16097903 10.1207/s15324796abm3001_4

[CIT0058] Stiggelbout, M., Hopman-Rock, M., Crone, M., Lechner, L., & Van Mechelen, W. (2006). Predicting older adults’ maintenance in exercise participation using an integrated social psychological model. *Health Education Research*, *21*(1), 1–14. 15980075 10.1093/her/cyh037

[CIT0059] Stults-Kolehmainen, M. A., & Sinha, R. (2014). The effects of stress on physical activity and exercise. *Sports Medicine*, *44*(1), 81–121. 24030837 10.1007/s40279-013-0090-5 PMC3894304

[CIT0060] Teixeira, P. J., Carraça, E. V., Markland, D., Silva, M. N., & Ryan, R. M. (2012). Exercise, physical activity, and self-determination theory: A systematic review. *International Journal of Behavioral Nutrition and Physical Activity*, *9*(1), 1–30. 10.1186/1479-5868-9-78 PMC3441783 22726453

[CIT0061] Trochim, W., & Kane, M. (2005). Concept mapping: An introduction to structured conceptualization in health care. *International Journal for Quality in Health Care*, *17*(3), 187–191. 15872026 10.1093/intqhc/mzi038

[CIT0062] Trochim, W. M. (1989). An introduction to concept mapping for planning and evaluation. *Evaluation and Program Planning*, *12*(1), 1–16.

[CIT0063] Turk, M. W., Yang, K., Hravnak, M., Sereika, S. M., Ewing, L. J., & Burke, L. E. (2009). Randomized clinical trials of weight-loss maintenance: A review. *The Journal of Cardiovascular Nursing*, *24*(1), 58–80. 19114803 10.1097/01.JCN.0000317471.58048.32 PMC2676575

[CIT0064] Wood, W., & Neal, D. T. (2016). Healthy through habit: Interventions for initiating & maintaining health behavior change. *Behavioral Science & Policy*, *2*(1), 71–83.

[CIT0065] Wood, W., Quinn, J. M., & Kashy, D. A. (2002). Habits in everyday life: Thought, emotion, and action. *Journal of Personality and Social Psychology*, *83*(6), 1281–1297. 12500811

