# Supplementary material for: Perspectives of health practitioners and adults who regained weight on predictors of relapse in weight loss maintenance behaviors: a concept mapping study
Source: Health Psychol Behav Med. 2021 Dec 26;10(1):22–40. doi: 10.1080/21642850.2021.2014332 (PMC8725894; doi:10.1080/21642850.2021.2014332)
Supplement: Supplemental Material [file RHPB_A_2014332_SM1932.docx]

**Supplementary file 1.**

**Questionnaire - Health professionals**

Q1. We ask you to write down as many ideas as possible regarding what, according to you, causes relapse in physical activity and dietary behavior. You can do this by answering the statements in front of the answer boxes below. Write down everything that comes to mind, there are no wrong ideas.

As some causes only apply to physical activity or to dietary behavior, but some causes apply to both physical activity and dietary behavior, there are separate answer boxes for:

1. The causes of relapse in dietary behavior
2. The causes of relapse in physical activity
3. The causes of relapse in physical activity and dietary behavior

As a reminder: by relapse we mean a situation in which an individual has started eating healthier and/or adopted a more physically active lifestyle, but over time falls back into previous more unhealthy habits. We are specifically interested in relapse in adults.

1. A relapse in dietary behavior is caused by … __________________
2. A relapse in physical activity is caused by … __________________
3. A relapse in physical activity and dietary
   behavior is caused by … __________________

Q2. What is your gender? ○ Male

○ Female

Q3. In which year were you born? __________________

Q4. What is your highest level of education? ○ Primary education

○ Lower general secondary education

○ Higher general secondary education

○ Pre-university education

○ Secondary vocational education

○ Bachelor’s degree

○ Master’s degree or Doctoral degree

Q5. What is your profession? ○ Dietitian

○ Weight loss consultant

○ Lifestyle coach

○ Other

Q6. How many years have you been working as a
 dietitian / weight loss consultant / lifestyle coach? ○ 0 – 5 years

○ 6 – 10 years

○ 11 – 15 years

○ 16 – 20 years

○ 21 – 25 years

○ 25 – 30 years

○ > 30 years

Q7. How many hours per week do you spend on average on patientcare?

○ Average amount of hours per week … __________________

○ I’m not involved in patientcare

Q8. What are the four digits of the postal code where
 you (mostly) work? __________________

**Questionnaire – Persons who regained weight**

Q1. We ask you to write down as many ideas as possible regarding what, according to you, causes relapse in physical activity and dietary behavior. You can do this by answering the statements in front of the answer boxes below. Write down everything that comes to mind, there are no wrong ideas.

As some causes only apply to physical activity or to dietary behavior, but some causes apply to both physical activity and dietary behavior, there are separate answer boxes for:

1. The causes of relapse in dietary behavior
2. The causes of relapse in physical activity
3. The causes of relapse in physical activity and dietary behavior

As a reminder: by relapse we mean a situation in which an individual has started eating healthier and/or adopted a more physically active lifestyle, but over time falls back into previous more unhealthy habits.

1. A relapse in dietary behavior is caused by … __________________
2. A relapse in physical activity is caused by … __________________
3. A relapse in physical activity and dietary
   behavior is caused by … __________________

Q2. What is your gender? ○ Male

○ Female

Q3. What is your age in years? __________________

Q4. What is your highest level of education? ○ Primary education

○ Lower general secondary education

○ Higher general secondary education

○ Pre-university education

○ Secondary vocational education

○ Bachelor’s degree

○ Master’s degree or Doctoral degree
